# Accuracy and reproducibility of a single‐pose image‐to‐robot registration method for mobile C‐arm cone beam CT guided histotripsy

**DOI:** 10.1002/acm2.70132

**Published:** 2025-05-31

**Authors:** Grace M. Minesinger, Paul F. Laeseke, Katrina L. Falk, Claire E. Hennen, Michael A. Speidel, Martin G. Wagner

**Affiliations:** ^1^ Department of Radiology University of Wisconsin‐Madison Madison Wisconsin USA; ^2^ Department of Medical Physics University of Wisconsin‐Madison Madison Wisconsin USA; ^3^ Department of Biomedical Engineering University of Wisconsin‐Madison Madison Wisconsin USA

**Keywords:** cone beam CT, histotripsy, registration

## Abstract

**Purpose:**

Histotripsy is a focal tumor therapy that utilizes focused ultrasound (US) to mechanically destroy tissue. To overcome visualization limitations of diagnostic US‐guidance, C‐arm cone beam CT (CBCT)‐guided histotripsy is being developed, for which a mobile C‐arm could increase accessibility. CBCT‐guided histotripsy uses a phantom with a helical fiducial pattern to determine the CBCT‐to‐histotripsy robot coordinate transformation. This study presents an image‐to‐robot registration method requiring only one phantom CBCT, evaluated for accuracy and reproducibility using a mobile C‐arm.

**Methods:**

The phantom is attached to a robotic arm (replacing the histotripsy transducer) and positioned at isocenter. A CBCT is acquired and image‐to‐robot registration performed by registering a digital model of the phantom to observed fiducials in CBCT coordinates. Registration was performed by one user (*n* = 8/day, 2 days) and by 12 different users (*n* = 4/day, 3 days) with fiducial registration errors (FREs) calculated. After each registration, the transducer was reattached to the robot and a treatment delivered in a multi‐layered, agar‐based phantom. Directional and target registration errors (TREs) were calculated as directional and Euclidean distances between planned and observed treatments. Directional error inter‐day differences for the single‐user experiment were evaluated for significance using 2‐tailed unpaired Student *t*‐tests. The effect of user variability on variability of FRE and directional error was evaluated for significance using 2‐tailed F‐tests.

**Results:**

Registrations yielded FRE of 0.12 ± 0.03 mm and TRE of 1.51 ± 0.83 mm. Targeting error significantly increased along the transducer's short axis between days (0.88 ± 0.60 vs 1.43 ± 0.18 mm, *p* = 0.025) for the single user, with a similar trend for the multi‐user experiment (1.45 ± 0.79, 2.70 ± 0.19, and 2.83 ± 0.40 mm). User variability, and thus robot pose variability, did not significantly affect variability of FRE or directional error.

**Conclusions:**

Mobile C‐arm CBCT‐guided histotripsy showed high accuracy with minimal yet nonnegligible TREs, consistent within but not between days, demonstrating that errors can be measured and accounted for, ideally near treatment day to maximize accuracy.

## 1 INTRODUCTION

Histotripsy is a nonthermal, nonionizing, and noninvasive focal tumor therapy, which recently received FDA approval for the treatment of liver tumors.^1^, ^2^ Histotripsy mechanically destroys tissue by emitting short, high amplitude ultrasound (US) pulses from a concave, multi‐element transducer. At the focal point, cavitation of endogenous tissue gas is induced, creating a bubble cloud that rapidly expands and collapses to mechanically rupture cells locally.^2^

The therapeutic transducer is attached to the end of a robotic arm with six degrees of freedom, which allows precise positioning and movement of the focal point during targeting and treatment.^3^ Histotripsy is currently guided by a diagnostic US probe that is coaxially aligned to the therapeutic transducer.^4^, ^5^, ^6^ While this approach works well in many cases, targeting tumors that are isoechoic with surrounding tissue or are blocked by intervening bone or gas is difficult and often not possible.^6^ However, even if the *diagnostic* beam path is obstructed, therapeutic US energy delivery is still possible in most cases, and tumors can still be treated with histotripsy.^7^ Thus, alternative imaging modalities are being investigated for guiding histotripsy treatment.

While magnetic resonance imaging (MRI) provides excellent soft tissue contrast and visibility of both tumors and histotripsy treatment zones,^8^, ^9^ MRI‐guided histotripsy requires MRI compatible hardware and involves longer scan times. Also, the closed bore of the MRI system does not provide enough space for the current histotripsy setup for an abdominal treatment, which requires a large water bath to rest on the patient for acoustic coupling. Computed tomography (CT), while ubiquitous in most clinical settings, also has a closed bore that is physically limiting in terms of size compatibility with the current histotripsy setup.

To provide a more open configuration for the current patient‐water bath setup and treatment, and to overcome US visualization limitations, C‐arm cone beam CT (CBCT) guided histotripsy has been developed.^10^, ^11^ Both fixed and mobile C‐arms with CBCT capabilities have been investigated for histotripsy image guidance, where fixed C‐arms are generally equipped with a higher powered x‐ray tube, have a larger field of view (FOV), and can produce images of higher quality.^12^ However, due to relatively high set‐up and maintenance costs and the need for a dedicated room for use, fixed C‐arms are not readily available in all centers or locations where histotripsy could theoretically be performed, such as surgical, interventional, or even outpatient centers.^13^ Mobile C‐arms are less expensive and can be shared between rooms and even buildings (with the minimum requirement of lead shielding), and thus could increase accessibility of this image guidance technique for histotripsy.^13^

CBCT guided histotripsy has been shown to yield high targeting accuracy in phantoms using both fixed and mobile C‐arm systems.^10^, ^11^ In previous work, an image‐to‐robot registration method (also known as hand‐eye calibration) was used to simultaneously estimate the transformations between coordinate systems of the CBCT volume, histotripsy robot, and histotripsy transducer.^10^ This method relies on 2D/3D pose estimation of the histotripsy transducer for multiple different robot poses from two simultaneously acquired 2D x‐ray images.^10^ Based on this work and the assumption that the relationship between histotripsy transducer and robotic arm is fixed and repeatable (due to the quick connect adapter used to mount the transducer), a modified approach was presented using a helical fiducial phantom.^11^ For this approach, a phantom with a helical pattern of stainless‐steel fiducials is attached to the robotic arm (instead of the transducer) and used to perform a similar image‐to‐robot registration.^11^ Like the previous approach, this requires multiple poses to simultaneously estimate the transformations between coordinate systems of the CBCT volume, histotripsy robot, and helical fiducial phantom using a single x‐ray image for each 2D/3D pose estimation. Subsequently, the known transducer to robotic arm relationship can be used to perform targeting after replacing the helical fiducial phantom with the histotripsy transducer. The helical fiducial phantom provides flexibility, enabling a single registration to support multiple transducers of varying geometries (e.g., different focal points). This enables targeting shallow and deep tumors in the same procedure without needing to re‐register the C‐arm for a different transducer, or without needing to increase the water bath volume to support larger gaps between the patient surface and transducer.

To build upon this work, we can also assume that the relationship between robotic arm and helical fiducial phantom is fixed and repeatable. This would allow estimating the transformation between CBCT and robot coordinate systems from a single image acquisition. In this work, a single‐pose registration method utilizing a 3D CBCT of the helical fiducial phantom is used to simplify the registration process. This simpler process can help streamline the integration of CBCT guidance with the current clinical histotripsy workflow. The purpose of this study was to evaluate the accuracy and repeatability of this registration approach for CBCT‐based histotripsy targeting using a mobile C‐arm.

## 2 MATERIALS AND METHODS

The goal of this image‐to‐robot registration is to determine the transformation between the local coordinate systems of the CBCT volume and histotripsy robot. In the proposed workflow, this is achieved using a single 3D CBCT of a helical fiducial phantom attached to the robotic arm, which is acquired prior to each histotripsy procedure without the patient present. Once the user positions the phantom in the CBCT FOV and acquires the CBCT, the transformation is automatically determined using the algorithm described in section 2.3. The following workflow assumes use of a mobile C‐arm, but registration can be performed with any C‐arm that has 3D CBCT capabilities. Once registration is performed, the relative positioning of the mobile C‐arm and histotripsy system must be fixed (i.e., wheels of both systems are locked) to maintain accurate registration. The helical fiducial phantom is replaced with the histotripsy transducer, whose focal point can be automatically aligned on any target using the registered robotic arm to deliver a treatment.

### 2.1 Transformations and notation

**FIGURE 1:**
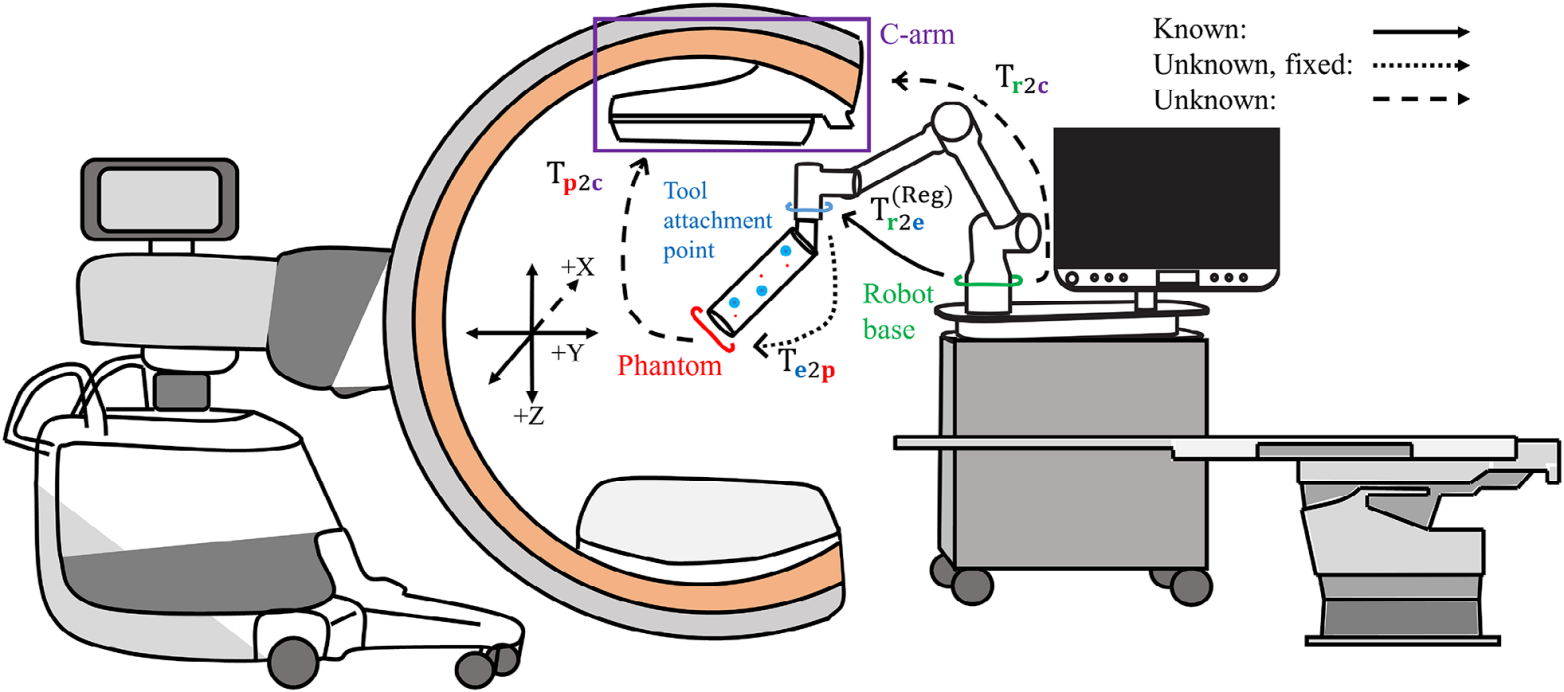
**Registration setup for mobile C‐arm cone beam CT (CBCT) guided histotripsy**. The CBCT coordinate system with directions X, Y, and Z, is shown in black. Transformations are shown between coordinate systems of the CBCT volume (c, purple), robot tool attachment point/end (e, blue), robot base (r, green), and helical fiducial phantom (p, red). Such transformations are either known, unknown but fixed (must be determined once), or unknown and change for every setup, indicated by curved arrows of different dash types. All transformations must be determined for a given robot pose to register the CBCT and histotripsy robot coordinate systems.

To determine this transformation between CBCT volume and histotripsy robot, transformations between local coordinate systems of *all* components of the CBCT guided histotripsy setup (see Figure 1) must be determined for a given robot pose. Components of interest include the base (r) and tool attachment point/end (e) of the robotic arm, the histotripsy transducer (h, not shown), helical fiducial phantom (p), and CBCT volume (c). Transformations between these components' local coordinate systems are either known, unknown but fixed (must be determined once), or unknown and change for every setup, indicated by curved arrows of different dash types in Figure 1. Transformations denoted as $T_{a2b}$ transform a given point in coordinate system “a” to a coordinate system “b”. Homogeneous coordinates will be used where any point, **a**, is represented by a 4 × 1 vector, and a transformation matrix, T, is represented by a 4 × 4 matrix consisting of scaling (s), rotation (r), and translation (**t**) components:

(1) $$a\;=\left[\begin{array}{c} a_{X}\\ a_{Y}\\ a_{Z}\\ 1 \end{array}\right]\;T=\left[\begin{array}{cccc} s_{X}r_{11} & s_{Y}r_{12} & s_{Z}r_{13} & t_{X}\\ s_{X}r_{21} & s_{Y}r_{22} & s_{Z}r_{23} & t_{Y}\\ s_{X}r_{31} & s_{Y}r_{32} & s_{Z}r_{33} & t_{Z}\\ 0 & 0 & 0 & 1 \end{array}\right]\;$$

Both histotripsy transducer and helical fiducial phantom can be easily attached to the robotic arm using a quick connect adapter (Quick Changer, OnRobot, Odense, Denmark). The single‐pose registration approach assumes that the attachment is repeatable, so given a fixed helical fiducial phantom geometry and fixed transducer model, the relationships between the attachments and the tool attachment point are fixed. Thus, transformations $T_{e2p}$ and $T_{e2h}$ can be calculated in one time registration processes using pose estimation from multiple robotic arm poses.^10^, ^11^ $T_{r2c}$ is the unknown transformation between robot base and CBCT coordinate systems, which changes every time the C‐arm or the histotripsy system is moved, and is thus the transformation of interest in this registration process.

Transformation $T_{r2e}$ is the current robotic arm pose and describes the relationship between the base and tool attachment point of the robot. Note that $T_{r2e}^{\left(Reg\right)}$ will be used to describe the robot pose during image‐to‐robot registration, and $T_{r2e}^{\left(Tx\right)}$ will be used to describe the robotic arm pose that aligns the histotripsy focal point with a target selected from a CBCT image volume. The latter can be calculated for any target location once $T_{r2c}$ has been estimated (once the CBCT volume and histotripsy robot have been registered).

### 2.2 Helical fiducial phantom

**FIGURE 2:**
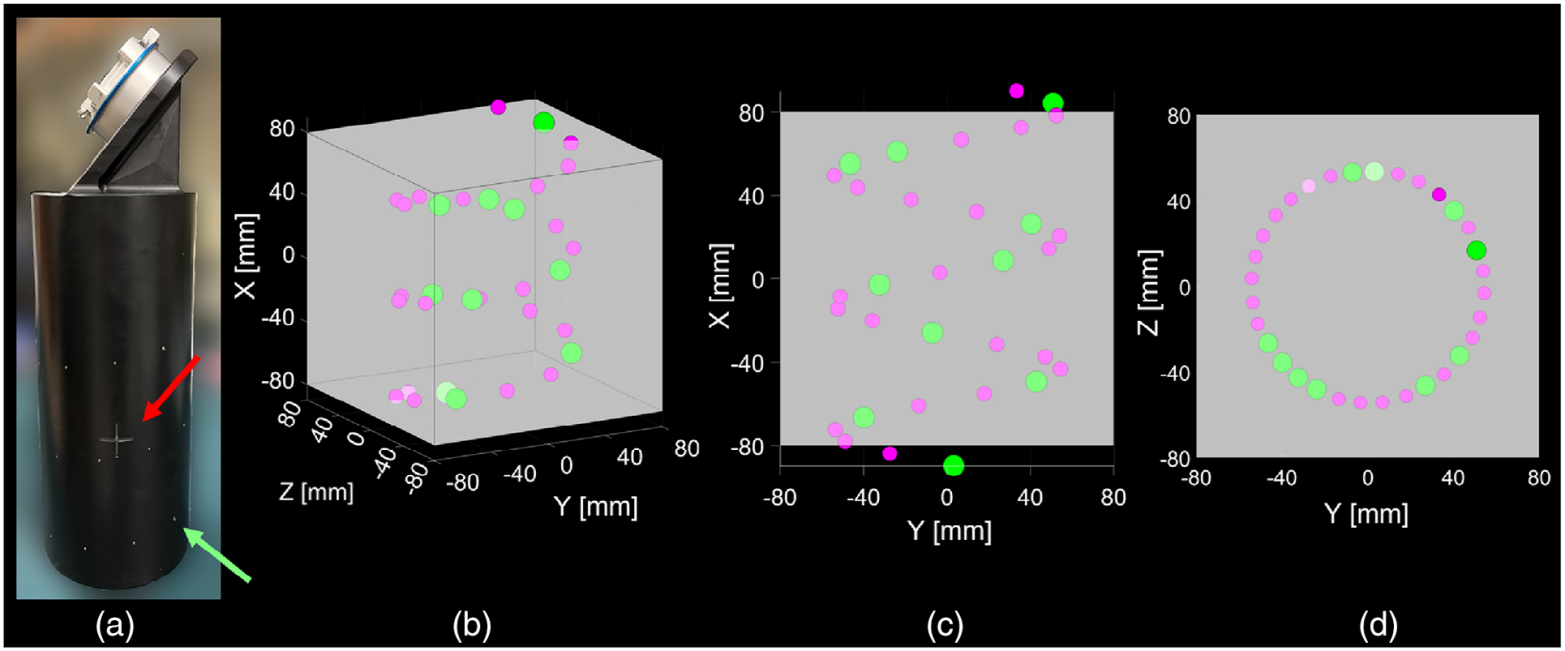
**Helical fiducial phantom used for image‐to‐robot registration**. (a): Image of the helical fiducial phantom with stainless steel fiducials (green arrow) and crosshairs (red arrow) used for C‐arm isocenter alignment. (b‐d): Renderings showing the phantom's full helical fiducial pattern with large (green) and small (magenta) fiducials, with an overlay (gray) of the (16 cm)^3^ FOV of the mobile C‐arm used in this work, perfectly centered about the phantom. This demonstrates the pattern of fiducials that encompass the full C‐arm FOV.

A helical fiducial phantom was designed to facilitate image‐to‐robot registration. The phantom consists of a PVC cylinder (diameter = 10.8 cm, length = 25.7 cm) with stainless steel fiducials placed in a helical pattern around its surface. Holes for fiducial placement were equidistantly positioned (3.2 cm apart) along a helical path (pitch = 6.2 cm). The helix consists of 22 small fiducials (1.5 mm diameter) and 10 larger fiducials (3 mm diameter). To provide a unique view in any 2D projection image or 3D CBCT, small and large fiducials were arranged in an asymmetric pattern. For ease of robotic arm manipulation in positioning the phantom in the C‐arm FOV, the phantom has visible crosshairs for C‐arm isocenter laser alignment (shown in Figure 2) and is angled at ∼50 degrees relative to the end of the robotic arm. The phantom was designed to provide fiducials that encompass as much of a C‐arm FOV as possible to increase precision in measuring the phantom's orientation. In smaller FOV scenarios, not all fiducials may be visible, but accurate registration is still possible. Thus, the minimum required number of fiducials to yield an accurate registration is approximated, and the maximum number of fiducials visible in the FOV of the C‐arm used in this study (Cios Spin 3D, Siemens Healthineers, Forchheim, Germany) is calculated, assuming the phantom is perfectly centered in the FOV with its long axis perfectly aligned with the rotational axis of the gantry.

### 2.3 Registration

For a given robot pose used for image‐to‐robot registration, $T_{r2e}^{\left(Reg\right)}$, the transformation from robot base to CBCT coordinates, $T_{r2c}$, is defined by the following relationship:

(2) $$\;T_{r2c}=T_{p2c}\;*T_{e2p}*T_{r2e}^{\left(Reg\right)}$$

$T_{p2c}$ is the only unknown transformation and defines the relationship between phantom and CBCT coordinate systems. Hence, $T_{p2c}$ can be defined by the transform that registers a digital model of the phantom's fiducials in phantom (p) space to fiducials in CBCT (c) space (denoted as “CBCT fiducials”) extracted from a CBCT volume. This fiducial registration approach minimizes the Euclidean distance between digital model fiducial centroids and the corresponding CBCT fiducial centroids.

The digital model of phantom fiducials was created as described in Wagner et al 2023.^11^ CBCT fiducials were segmented from the CBCT volume using a global threshold τ corresponding to the 99.99th percentile value in the CBCT. The percentile was determined based on the number of expected fiducial voxels in the image. A connected component analysis was performed to identify individual fiducials and each fiducial's geometrical centroid and equivalent diameter. Fiducials were then grouped into small and large fiducials using a threshold of 2.25 mm.

Correspondence between all large digital model fiducials, $f_{j}^{\left(\mathrm{model}\right)}$, and all large CBCT fiducials, $f_{i}^{\left(\mathrm{CBCT}\right)}$, was determined by calculating the Euclidean distances from each large fiducial to all other large fiducials within the same fiducial set (CBCT or model). For each fiducial $f_{i}^{\left(\mathrm{CBCT}\right)}$, the corresponding fiducial $f_{j}^{\left(\mathrm{model}\right)}$ was chosen to minimize

(3) $$\begin{array}{cc} & d\;\left(i,j\right)\\ & =\sum_{k=1}^{m-1}\min_{j^{\prime }}\left(∥f_{i}^{\left(\mathrm{CBCT}\right)}-f_{{\left(i+k\right)}_{m}}^{\left(\mathrm{CBCT}\right)}{∥}_{2}-∥f_{j}^{\left(\mathrm{model}\right)}-f_{j^{\prime }}^{\left(\mathrm{model}\right)}{∥}_{2}\right)\; \end{array}$$

using a brute force approach, where m is the number of large fiducials visible in the CBCT volume. The set of m corresponding coordinates are related by the affine transformation $T_{p2c}$ as follows:

(4) $$\begin{array}{cc} & T_{p2c}*\;\left[\begin{array}{cccc} f_{1}^{\left(\mathrm{model}\right)} & f_{2}^{\left(\mathrm{model}\right)} & \text{…} & f_{m}^{\left(\mathrm{model}\right)} \end{array}\right]\\ & =\left[\begin{array}{cccc} f_{1}^{\left(\mathrm{CBCT}\right)} & f_{2}^{\left(\mathrm{CBCT}\right)} & \text{…} & f_{m}^{\left(\mathrm{CBCT}\right)} \end{array}\right]\; \end{array}$$

Given this relationship, $T_{p2c}$ was approximated using least square estimation via complete orthogonal decomposition of the matrix of $f_{j}^{\left(\mathrm{model}\right)}$ coordinates.^14^ $T_{r2c}$ was then initialized using its relation to this $T_{p2c}$ estimation (Equation 2).

An iterative optimization approach was then used to further refine the estimated transform $T_{r2c}$. To this end, a cost function was defined which calculates the root mean square error (RMSE) of Euclidean distances between all CBCT fiducials (small and large) and the closest model fiducials after applying transform $T_{p2c}$. Iterations continued until a maximum of 2000 iterations, the change in cost function value (RMSE) was less than 10^−3^ mm, or the step size was less than 10^−3^ mm.

Once $T_{r2c}$ has been estimated (image‐to‐robot registration performed), the robotic arm pose, $T_{r2e}^{\left(Tx\right)}$, to align the transducer focal point on any location in CBCT space, (**x**), is calculated using

(5) $$\;T_{r2e}^{\left(Tx\right)}={\left(T_{e2h}\right)}^{-1}\;*{\left[\begin{array}{cccc} & & & x_{X}\\ & R_{h2c} & & x_{Y}\\ & & & x_{Z}\\ 0 & 0 & 0 & 1 \end{array}\right]}^{-1}*T_{r2c}$$

where $R_{h2c}$ is the 3 × 3 rotational transformation between the transducer and the CBCT coordinate system, representing angular tilts (if any) of the transducer to provide an optimal pathway for energy delivery to the target. $T_{e2h}$ could be determined from pose estimation with the histotripsy transducer^12^ or determined directly from computer aided design (CAD) models for manufacturing.

### 2.4 Experimental setup

In practice, many variables can influence the accuracy of CBCT‐based histotripsy targeting, including the accuracy of the registration, positioning of the helical fiducial phantom, repeatability of the CBCT FOV, the accuracy of the assumed $T_{e2h}$ (especially when using CAD model as reference), and the repeatability of the transducer and phantom attachment to the robotic arm. The experiments described in this section aim to evaluate the repeatability of the image‐to‐robot registration and resulting targeting accuracy within the same day, over multiple days, and across multiple users.

In a “single‐user” experiment, image‐to‐robot registration was repeated multiple times (*n* = 16) by one user with trials performed on two different days (*n* = 8 per day). Additionally, a “multi‐user” experiment was performed, where image‐to‐robot registration was repeated multiple times (*n* = 12), each time by a different user, with trials performed on three different days (*n* = 4 per day). The multi‐user experiment was performed to evaluate the effect of variability in positioning the fiducial phantom in the FOV on registration and targeting accuracy. For the single‐ and multi‐user experiments, the same fiducial phantom, C‐arm, histotripsy system, and histotripsy transducer were used.

Pose estimation to determine $T_{e2p}$ was performed once prior to all experiments, and $T_{e2h}$ was estimated directly from the transducer's CAD model. The C‐arm was re‐positioned once at the start of each day of trials and the wheels were locked. Before each registration instance, the user re‐positioned the histotripsy system and locked the wheels, attached the helical fiducial phantom to the robotic arm using the quick connect adapter, and used the robotic arm to position the phantom in the C‐arm FOV, aligning the C‐arm lasers with the center of the phantom (marked by crosshairs, see Figure 2). A 3D CBCT was then acquired using the highest dose protocol (60 s rotation, 400 projections) to maximize image quality, and was reconstructed with a standard kernel. CBCT fiducials were then segmented and registered with the digital model fiducials using the method described in section 2.3 to determine $T_{r2c}$, hence registering CBCT and histotripsy robot coordinate systems. In all registration instances, both small (1.5 mm) and large (3 mm) fiducials were utilized to estimate the transformation $T_{r2c}$. Once registration is performed, neither system is moved (wheels were kept locked) because registration accuracy relies on the spatial relationship between C‐arm and histotripsy robot. The following processes were performed identically across single‐ and multi‐user experiments, all performed by the same single user.

To evaluate the targeting accuracy of this approach, after each registration instance in each trial, a histotripsy bubble cloud (a few mm in size, no translation through a treatment volume) was created in a targeted agar‐based phantom and maintained for approximately 20 s to serve as a “bubble cloud treatment”. The phantoms consisted of alternating layers of agar (∼3.5 mm thick, 1.5 % Agar #12177, Fischer Scientific, St. Louis, MO) and agar with barium (∼1 mm thick, 6%, Barium Sulfate Powder, Carolina Biological Supply Company, Burlington, NC). Alternating layers served as CBCT contrast: before treatment, distinct layers were visible in CBCT volumes due to differences in x‐ray attenuation, and after treatment, layers mixed to create an area of homogeneous attenuation, distinguishable from surrounding, intact layers.^15^

For each bubble cloud treatment, a multi‐layered phantom was placed in a degassed water bath for acoustic coupling, and a pre‐treatment CBCT was acquired (30 s rotation, 400 projections) and reconstructed with a standard kernel. The 30 s acquisition was used here to mimic an acquisition that would be used clinically for targeting histotripsy, as the shorter duration minimizes dose as well as scan time to enable a more feasible breath‐hold. Using the CBCT as reference, a target point in the phantom was chosen using a custom treatment planning software (ImFusion GmbH, Munich, Germany). $T_{r2e}^{\left(Tx\right)}$ was then determined using Equation 5. The robotic arm (UR5e, Universal Robots, Odense, Denmark) automatically moved to the prescribed pose, and the 700 kHz multielement histotripsy transducer (research system, Histosonics, Inc., Plymouth, MN) delivered the bubble cloud treatment.

For each treatment, the voltage used was recorded as a percent of the system's maximum voltage. Acoustic aberration correction, a process typically performed clinically to account for bubble cloud shifts due to speed of sound heterogeneities, was not performed here because the beam paths here mostly intersected with water which has a constant speed of sound, and minimal offsets were expected. After delivering each treatment, a final post‐treatment CBCT was acquired of the phantom using the same acquisition parameters as the pre‐treatment image. And because CBCTs are acquired either before or after histotripsy treatments, the two mechanisms are not expected to interfere/interact simultaneously with each other. To facilitate the targeting accuracy evaluation, neither the multi‐layered phantom, water bath setup, nor the mobile C‐arm was moved between pre‐ and post‐treatment scans. After the final post‐treatment CBCT was acquired, the histotripsy system was moved again, so it could be re‐positioned for the next registration and targeting trial.

### 2.5 Analysis

Tube parameters (kV, mAs) were recorded for each image acquisition. The number of small and large fiducials detected in each CBCT scan of the helical fiducial phantom was recorded. Accuracy of each fiducial registration instance, which influences the resulting image‐to‐robot registration, is denoted as the fiducial registration error (FRE). This is calculated as the root mean square error of Euclidean distance between detected CBCT fiducials and corresponding digital model fiducials registered from the phantom coordinate system to the CBCT coordinate system by the transform $T_{p2c}$.

Areas homogenized from bubble cloud treatments in post‐treatment CBCT images of multi‐layered phantoms were semi‐automatically segmented in 3D Slicer (Slicer 5.0.3) using a modified version of the grow‐cut algorithm.^16^ To remove noise, only the largest object in each segmentation was extracted using connected component analysis, and then segmentations were smoothed with a median filter. Additionally, a morphological filter was applied to fill any holes in the segmentation (kernel sizes = 1 mm). Targeting accuracy was measured as the distance between planned target and measured bubble cloud treatment centroid, where Euclidean distance errors (Target Registration Error, TRE) and directional errors in X, Y, and Z were recorded.

The difference in directional targeting errors in each spatial dimension between days in the single‐user experiment was evaluated for statistical significance using 2‐tailed unpaired Student *t*‐tests. The effect of user variability on the variability of FRE and directional targeting error was evaluated for statistical significance. To this end, 2‐tailed F‐tests were performed to evaluate whether the variance across multiple users was larger than the variance for a single user. This was done for both FRE and targeting error in each direction. To remove the bias for individual days, the average metric for each day was subtracted from all individual measurements prior to performing the F tests. Similarly, the effect of user variability on robot positioning variability was evaluated for statistical significance. To this end, 2‐tailed F‐tests were performed to evaluate whether the robot pose variance across multiple users was larger than the variance for a single user. This was done for the position of the robotic arm in X, Y, and Z, the rotational axis components in X, Y, and Z, and the angle of rotation, for a total of seven robot pose statistical tests. A statistical significance level of α = 0.05 was used for all hypothesis tests. Because the seven components of the robot pose may be correlated, however, the Benjamini–Hochberg procedure was performed for the robot pose tests to reduce the false discovery rate by lowering α for the lowest resulting *p*‐values.

## 3 RESULTS

### 3.1 Fiducial registration reproducibility

All metrics are reported as the average ± standard deviation. C‐arm tube parameters from scans of the helical fiducial phantom were 110.916 ± 0.005 kV and 34 ± 2 mAs. If the phantom is perfectly centered in the Cios Spin's (16 cm)^3^ FOV using isocenter lasers for alignment, only 8/10 (80.0%) large fiducials, 20/22 (∼90.9%) small fiducials, and thus a total of 28/32 (87.5%) fiducials can be detected, where the truncated fiducials were only on the top and bottom of the phantom. Based on the phantom's fiducial pattern, only 4/10 large fiducials (40%) must be detected to uniquely identify each fiducial.

**FIGURE 3:**
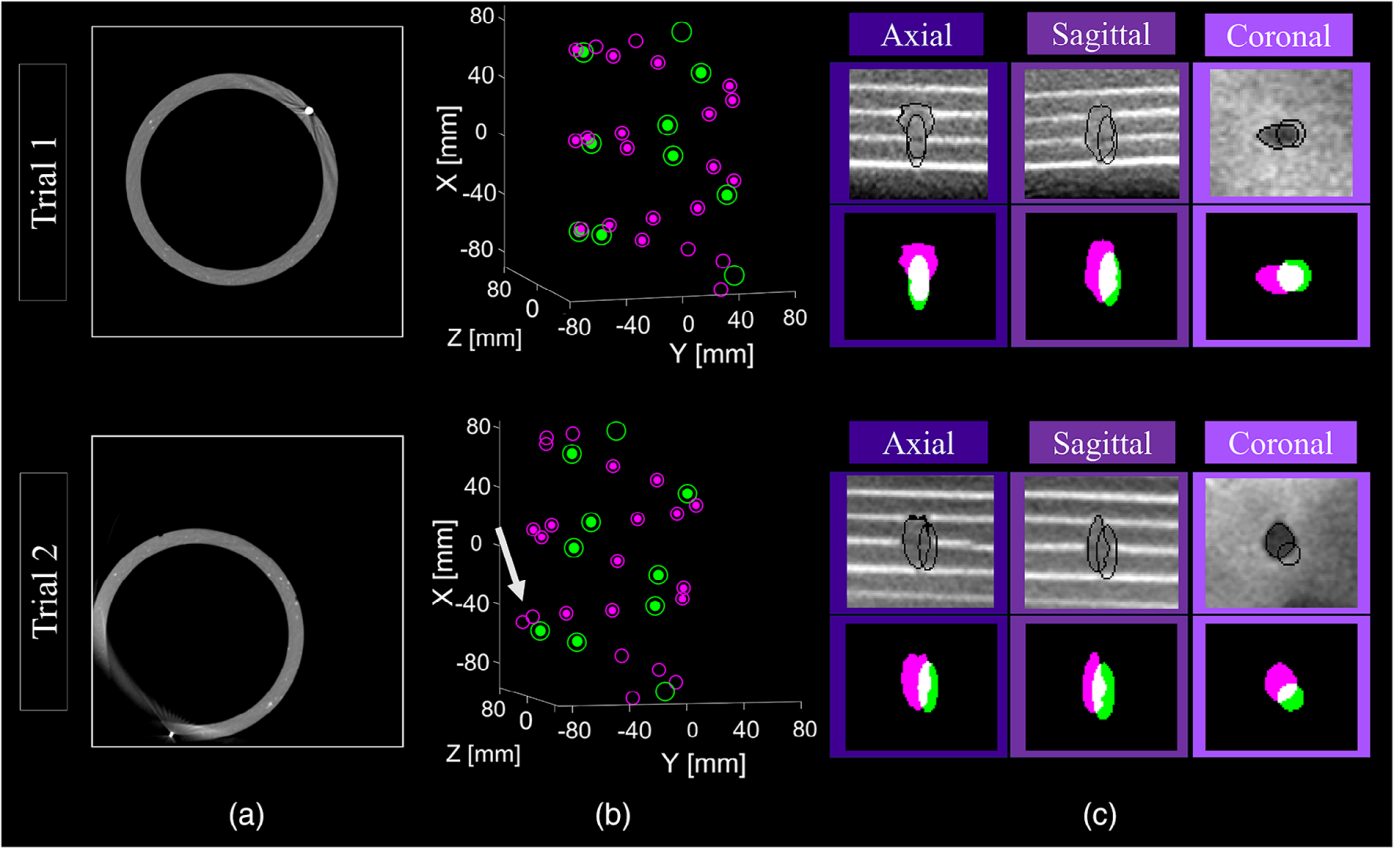
**The process of image‐to‐robot registration and histotripsy targeting accuracy evaluation**. Two trials are shown here (trial 1 from the single‐user experiment; trial 2 from the multi‐user experiment). (a) Axial slices of 3D cone beam CT (CBCT) images of the helical fiducial phantom. (b) 3D renderings of large (green) and small (magenta) phantom fiducials, demonstrating successful registrations of digital model fiducials (open circles) to CBCT fiducials (closed circles). Fiducial registration in both trials were successful even despite the inevitable axial truncation of the phantom, and the avoidable lateral truncation in trial 2 which led to two additional undetected fiducials (white arrow). (c) 2D CBCT slices showing the targeting error as the distance between planned and observed histotripsy bubble cloud treatments in a multi‐layered agar phantom. For each trial, the top row shows segmentations outlined in black over the grayscale image, and the bottom row shows overlap (white) of observed (magenta) and planned (green) treatments. This demonstrated minimal but measurable CBCT guided histotripsy targeting errors.

All users successfully positioned the histotripsy system and aligned the helical fiducial phantom in the C‐arm FOV. A total of 24.5 ± 1.0 fiducials out of the possible 28 (87.5 ± 3.6%) were detected in each CBCT scan. Of those, 7.8 ± 0.4 large fiducials out of the possible 8 (97.5 ± 5.3%) and 16.7 ± 1.0 small fiducials out of the possible 20 (83.4 ± 5.0%) were detected. Following registration of digital model fiducials and CBCT fiducials, the FRE was 0.12 ± 0.03 mm (see examples in Figure 3). The multi‐user experiment resulted in robot registration poses with a significantly greater variance than those performed by the single user for all components of the robot pose except for the angle of rotation. Results for positions in X, Y, Z, rotation axis components in X, Y, Z, and the angle of rotation were F(15,11) = 0.25, 0.05, 0.06, 0.29, 0.03, 0.20, 0.75, *p* = 1.3e‐2, 1.9e‐6, 2.3e‐6, 2.6e‐2, 1.5e‐8, 5.4e‐3, 5.9e‐1, respectively. User variability showed no significant effect on FRE variability, F(15,11) = 0.50, *p* = 0.215.

### 3.2 Targeting accuracy

All bubble cloud treatments were successfully delivered and visible on post‐treatment CBCT images. C‐arm tube parameters from pre‐ and post‐treatment scans of the multi‐layered phantom were 110.97 ± 0.03 kV and 620 ± 110 mAs. The reported histotripsy system voltage used was 23.1 ± 2.9%. Across all days of single‐ and multi‐user experiments, unsigned average directional targeting errors were 1.70 ± 0.82, 0.92 ± 0.49, and 1.94 ± 0.78 mm in X, Y, and Z directions, respectively (Tables 1 and 2). The signed average targeting errors were similar (1.65 ± 0.87, 0.90 ± 0.52, and 1.94 ± 0.78 mm). The average TRE was 1.51 ± 0.83 mm. TREs and unsigned and signed directional targeting errors for each day of trials are shown in Tables 1 and 2 for single‐ and multi‐user experiments, respectively.

**TABLE 1:** **Single‐user cone beam CT image‐to‐histotripsy robot registration and subsequent targeting accuracy evaluation in a phantom**.

				Measured Targeting Errors [mm]			
Day	FRE [mm]	Voltage (%)	Mean ± SD	X	Y	Z	TRE
1	**0.13 **± 0.03	**21.8 **± 1.0	Unsigned	**0.96 **± 0.42	**0.88 **± 0.41	**2.63 **± 0.44	**1.49 **± 0.92
			Signed	**0.88 **± 0.60	**0.88 **± 0.41	**2.63 **± 0.44	n/a
2	**0.12 **± 0.01	**26.4 **± 0.9	Unsigned	**1.43 **± 0.18	**0.73 **± 0.42	**2.22 **± 0.62	**1.46 **± 0.75
			Signed	**1.43 **± 0.18	**0.68 **± 0.51	**2.22 **± 0.62	n/a
Mean ± SD	**0.12 **± 0.02	**24.1 **± 2.6	Unsigned	**1.19 **± 0.39	**0.81 **± 0.41	**2.43 **± 0.56	**1.48 **± 0.83
			Signed	**1.15 **± 0.51	**0.78 **± 0.46	**2.43 **± 0.56	n/a

*Note*: Results of the single‐user experiment, where a single user registered image and robot coordinate systems (*n* = 8 per day for 2 days) and subsequent histotripsy treatments were delivered in a phantom to measure targeting accuracy. Mean ± standard deviation (SD) is shown for each metric. Results for each day of treatment show fiducial registration error (FRE), histotripsy treatment voltages, measured directional targeting errors (−Z = toward transducer), and Euclidean distance targeting errors (Target Registration Error, TRE).

**TABLE 2:** Multi‐user cone beam CT image‐to‐histotripsy robot registration and subsequent targeting accuracy evaluation in a phantom.

				Measured Targeting Errors [mm]			
Day	FRE [mm]	Voltage (%)	Mean ± SD	X	Y	Z	TRE
1	**0.15 **± 0.06	**25.5 **± 1.7	Unsigned	**1.45 **± 0.79	**1.1**4 ± 0.75	**1.54 **± 0.38	**1.38 **± 0.63
			Signed	**1.45 **± 0.79	**1.1**4 ± 0.75	**1.54** ± 0.38	n/a
2	**0.13 **± 0.03	**20.3 **± 1.5	Unsigned	**2.70 **± 0.19	**1.1**8 ± 0.72	**1.17 **± 0.66	**1.68 **± 0.91
			Signed	**2.70 **± 0.19	**1.18 **± 0.72	**1.17 **± 0.66	n/a
3	**0.12 **± 0.00	**19.8 **± 0.5	Unsigned	**2.83 **± 0.40	**0.88** ± 0.18	**1.14 **± 0.47	**1.62 **± 0.96
			Signed	**2.83 **± 0.40	**0.88 **± 0.18	**1.14 **± 0.47	n/a
Mean ± SD	**0.13 **± 0.04	**21.8 **± 3.0	Unsigned	**2.32 **± 0.80	**1.07 **± 0.57	**1.28 **± 0.50	**1.56 **± 0.83
			Signed	**2.32 **± 0.80	**1.07** ± 0.57	**1.28 **± 0.50	n/a

*Note*: Results of the multi‐user experiment, where different users registered image and robot coordinate systems (*n* = 4 per day for 3 days) and subsequent histotripsy treatments were delivered in a phantom to measure targeting accuracy. Mean ± standard deviation (SD) is shown for each metric. Results for each day of treatment show fiducial registration error (FRE), histotripsy treatment voltages, measured directional targeting errors (−Z = toward transducer), and Euclidean distance targeting errors (Target Registration Error, TRE).

The difference between day 1 and day 2 targeting errors in the X direction (along the short axis of transducer) from the single‐user experiment (0.55 ± 0.64 mm) was statistically significant, t(14) = 2.51, *p* = 0.025, with a 95% confidence interval of [0.08 mm, 1.03 mm]. Differences between day 1 and day 2 targeting errors in Y (−0.20 ± 0.57 mm) from the single‐user experiment were not statistically significant, t(14) = −0.86, *p* = 0.402. Similarly, differences in Z errors (−0.41 ± 0.66 mm) were not statistically significant, t(14) = −1.52, *p* = 0.151. User variability showed no significant effect on targeting error variability in the X, Y, or Z directions, F(15,11) = 0.81, 0.66, and 1.24, *p* = 0.696, 0.441, and 0.727, respectively.

## 4 DISCUSSION

This study presents a CBCT image‐to‐robot registration method that enables automated CBCT‐based targeting for histotripsy treatments. The proposed registration method builds upon previous work in that it only requires a single 3D CBCT acquisition of the helical fiducial phantom before treatment, providing a more streamlined workflow for everyday use. The transformation between histotripsy robot and CBCT coordinate systems is determined by registering the helical fiducial phantom fiducials in its own coordinate system to those detected in the CBCT volume. Registration can be done any time prior to a histotripsy procedure before the patient is in the room, as long as the C‐arm and histotripsy systems are fixed in place until treatment. While the systems cannot be moved after registration, the table can still be translated out of the FOV to provide easier access and greater flexibility in positioning the patient on the table for therapy. When it is time to deliver a histotripsy treatment, the fiducial phantom that was attached to the robot is simply replaced with the transducer. Accuracy and reproducibility of this approach using a mobile C‐arm was evaluated by repeated registrations performed by different users and over multiple days. Targeting accuracy for each registration trial was evaluated by comparing the location of a target in an agar‐based multi‐layered phantom and the observed histotripsy bubble cloud treatment centroid.

All fiducial registrations yielded FREs each less than 0.3 mm, and there was no significant effect of user variability on the variability of FRE or directional targeting error. And since there *was* a significant effect of user variability on the variability of robot poses, slight variations in robot poses to position the phantom for registration has no suspected influence on the targeting accuracy of CBCT guided histotripsy. The top and/or bottom of the phantom was truncated in all images, but in only one trial, the side of the helical fiducial phantom was truncated due to poor centering (trial 2 of Figure 3), and thus the fewest number of fiducials was detected out of all other trials (21 total = 7 large + 14 small). Even in this nonideal scenario, fiducial registration accuracy was still adequate (FRE = 0.23 mm). Importantly, the FRE alone cannot determine if the registration succeeded: if only a single fiducial is detected in the CBCT, it can be registered to any phantom fiducial and yield an FRE of 0 without recognizing the helical fiducial phantom's pattern. Thus, it was estimated that a minimum of four large fiducials is necessary to detect the helical fiducial pattern, a criterion that was met in all trials (minimum detected, large = 7). This suggests that the fiducial registration, and hence image‐to‐robot registration, is robust to varying positions and FOV‐coverage of the helical fiducial phantom, which are inevitable to occur when users of varying experience position the phantom in the FOV and when using C‐arms with smaller FOVs. And while only one FOV and one phantom size was investigated in this study, theoretically any FOV size and phantom can be used as long as (1) the phantom has enough fiducials to provide a unique pattern in a given FOV size, and (2) the phantom is small enough to provide some flexibility when positioned slightly off‐isocenter.

Targeting accuracy was consistently high, with treatments delivered within 2 mm of the target on average, and a standard deviation within 1 mm. In the single‐user experiment, a significant increase in targeting error along the transducer's short axis (+X) was observed between days 1 and 2. A similar increased targeting error along +X was observed in the multi‐user experiment between days. This suggests that targeting errors are consistent on a given day, but may change over the span of multiple days, and thus should be measured and accounted for as close to the day of treatment as possible.

The consistency of observed daily targeting errors also indirectly demonstrated that CBCT representation of physical space (the FOV) is highly reproducible between scans on a given day. If the FOV was not reproducible between scans, the relationship between C‐arm FOV and histotripsy system would change between scans, rendering the pre‐determined $T_{r2c}$ inaccurate, which would have resulted in lower and more variable targeting accuracy. C‐arm FOV reproducibility depends on a variety of factors, including mechanical stability as well as its degrees of freedom (DOF) relative to its base and the room. More mechanically stable C‐arms and those with fewer DOF, such as fixed C‐arms (floor‐mounted systems), are expected to provide higher reproducibility. Thus, reproducibility especially needed to be evaluated for the mobile C‐arm due to its many DOF and mobile base, where gantry flex inconsistencies and mechanical imperfections could contribute to variations in the C‐arm's position in space as well as parameters relative to the C‐arm. In another study, parameters relative to the C‐arm for the Cios Spin were found to vary during a single rotation but varied reproducibly across multiple scans.^17^ Reproducibility of the C‐arm's position in space, however, needed to be studied, and now has been indirectly observed to be consistent across multiple scans on a given day for use in CBCT guided histotripsy.

Limitations of this work include the lack of a known cause or causes for the observed targeting errors and their changes over time. These could be due to errors involved in CBCT image‐to‐robot registration or small deviations in the bubble cloud location relative to the histotripsy robot. While targeting errors were observed, they can be measured and compensated for via a calibration step.^18^  And while the stability of each registration was not evaluated long‐term, the proposed registration process is expected to be performed just prior to each histotripsy treatment to ensure the histotripsy system and C‐arm are not moved between registration and treatment.

Another limitation in this study was that fiducial registration and histotripsy targeting accuracy and reproducibility were only evaluated with a single C‐arm. Reproducibility of a different C‐arm's representation of physical space, which may be specific to each model and manufacturer, may not be guaranteed and should be evaluated for use in CBCT guided histotripsy. Different C‐arms may have smaller FOVs, but image‐to‐robot registration could still be performed accurately if at least four phantom fiducials can fit in the FOV. The presented registration process would still be identical for all C‐arms with CBCT capabilities.

Also, the proposed process of registration and treatment must be performed while following radiation safety guidelines (lead shielding and personal protection)—a consideration unnecessary for the ultrasound‐guided workflow, but one that is achievable in many clinical settings. Two contrast‐enhanced CBCTs would be acquired during the procedure, the first for visualizing the tumor for targeting, and the second to visualize the treatment and confirm adequate margins, resulting in a cumulative radiation dose comparable to other interventional procedures.^19^ Also, the proposed method was validated using phantoms to provide a controlled environment for repeatability testing, and was not validated clinically in this study. Clinical validation of *fixed* C‐arm CBCT guided histotripsy has been investigated^19^ and clinical validation of *mobile* C‐arm CBCT guided histotripsy is a crucial next step planned for future work.

## 5 CONCLUSION

CBCT guided histotripsy overcomes US‐guidance limitations to increase the scope of patients that could be treated with histotripsy. Compared to using a fixed C‐arm, a mobile C‐arm would provide a more accessible, flexible, and yet still robust and reproducible option for histotripsy targeting. This work presented a one‐step image‐to‐robot registration method, which was shown to be accurate, reproducible, and simple, promising a streamlined integration into the clinical workflow of histotripsy treatments. Thus, this study supports further investigation of the clinical feasibility of histotripsy guidance with a mobile C‐arm.

## AUTHOR CONTRIBUTIONS

All authors helped draft and/or revise this manuscript. All authors approved the final version of this manuscript and agree to be accountable for all aspects of this work. Author G.M. led the conception and design of this work, as well as the acquisition, analysis, and interpretation of data for this work. Author K.F. contributed to the concept and design by discussing experimental approaches for delivering histotripsy in phantoms. Author C.H. contributed to the acquisition of data for the work by creating phantoms and helping acquire cone beam CT images and delivering histotripsy treatments. Author P.L. contributed to the concept and interpretation of this work through discussing clinical implications and impacts of using a mobile C‐arm to guide histotripsy. Author M.S. contributed to the design of this work through discussions of experimental design to demonstrate reproducibility. Author M.W. contributed to the work's concept, design, and interpretation by developing the registration method and advising methods to acquire data and perform statistical tests that demonstrate reproducibility.

## CONFLICT OF INTEREST STATEMENT

Author P.L. has a financial relationship with HistoSonics (Shareholder and Consultant); J&J/Ethicon/NeuWave (Consultant); Siemens (sponsored research). Authors P.L., M.S., and M.W. have sponsored research agreements with Siemens Healthineers. Author M.W. is a consultant and receives research support from HistoSonics, Inc. Author K.F. is a consultant for HistoSonics, Inc. Authors G.M. and C.H. do not have anything to disclose.

## Data Availability

Authors will share data upon request to the corresponding author.
